# Motivational Interviewing and Return to Work for People with Musculoskeletal Disorders: A Systematic Mapping Review

**DOI:** 10.1007/s10926-020-09892-0

**Published:** 2020-04-30

**Authors:** Fiona Aanesen, Rigmor Berg, Ida Løchting, Alexander Tingulstad, Hedda Eik, Kjersti Storheim, Margreth Grotle, Britt Elin Øiestad

**Affiliations:** 1 Department of Physiotherapy, Oslo Metropolitan University, Oslo, Norway; 2 Norwegian Institute of Public Health, Oslo, Norway; 3 Research and Communication Unit for Musculoskeletal Health (FORMI), Oslo University Hospital, Oslo, Norway

**Keywords:** Motivational interviewing, Return to work, Musculoskeletal diseases, Sick leave, Systematic review

## Abstract

*Purpose* There is limited knowledge about motivational interviewing (MI) for people on sick leave with musculoskeletal disorders. Hence, our objective was to investigate *what research on MI as a method to facilitate return to work for individuals who are on sick leave due to musculoskeletal disorders exists, and what are the results of the research? Methods* We systematically searched MEDLINE, PsycINFO, EMBASE, Cochrane Library, CINAHL, Web of Science, Sociological Abstracts, Epistemonikos, SveMed + and DARE & HTA (covering 1983 to August 2019). We also searched the MINT bulletin and relevant web pages. Eligibility criteria: empirical studies investigating MI and return to work for people with musculoskeletal disorders. Two authors independently screened the records, critically appraised the studies and charted the data using a data extraction form. *Results* The searches identified 1264 records of which two studies were included. One randomized controlled trial (RCT) found no effect of MI on return to work for disability pensioner with back pain (n = 89, high risk of bias), while a cluster RCT found that MI increased return to work for claimants with chronic musculoskeletal disorders (n = 728, low risk of bias). *Conclusions* This mapping review identified a huge gap in research on MI to increase return to work for individuals with musculoskeletal disorders. *Registration* Current Research Information System in Norway, project id: 635823 (https://app.cristin.no/projects/show.jsf?id=635823).

**Electronic supplementary material:**

The online version of this article (10.1007/s10926-020-09892-0) contains supplementary material, which is available to authorized users.

## Introduction

Musculoskeletal disorders affecting joints, bone and soft tissues are the leading cause of disability worldwide [1]. Neck and back pain, osteoarthritis and inflammatory diseases, osteoporosis, bursitis, tendonitis and fibromyalgia are most common [2]. The disorders often have fluctuating symptoms which can reduce work ability [3]. For people living with musculoskeletal disorders long periods of sickness absence can be detrimental for wellbeing and hinder return to work, while work and activity can aid recovery [4].

Work participation is dependent upon several social, workplace-related and individual factors [5, 6]. Many different coordinated return to work programmes have been developed to address these factors such as tailored work rehabilitation, case management and collaborative care. These programmes include an assessment of the workers’ needs in order to make a return to work plan. The worker can receive a variety of tailored interventions such as medical interventions, education, workplace ergonomics and case management to assist in their return to work. The interventions are usually coordinated and provided by different professions such as physiotherapists, occupational therapists, social workers, psychologists and physicians. Some of the interventions also involve the employer [7]. A Cochrane review from 2017 investigating the effects of return to work coordination programmes versus usual practice on return to work outcomes, including 14 RCTs, showed small to no benefits of such programmes. The evidence from the review was low to moderate due to imprecision and substantial heterogeneity between the studies [7].

Motivational interviewing (MI) has been suggested as a suitable method in vocational rehabilitation [8–10]. MI is a person-centred counselling style for addressing ambivalence and strengthen motivation, by exploring the person’s own reasons for change [11]. Miller and Rollnick developed MI for the treatment of addictions and define it as ‘a collaborative, goal-oriented style of communication with particular attention to the language of change’ [11] (p. 29). MI is associated with small to medium effect sizes across a variety of behaviour outcomes [11]. The method has been used to support behavioural change for people with different conditions, including musculoskeletal disorders [12] and chronic pain [13]. MI could be a suitable tool to improve working alliance between caseworkers and people on sick leave [14]. This might be especially important for people suffering from unspecific musculoskeletal disorders who often face mistrust and scepticism related to their health problems [3, 15, 16]. The results from a systematic review from 2017 investigating the effectiveness of MI to facilitate return to work suggested that MI may be an effective intervention, although the authors could not draw any conclusions due to few studies and low quality of the evidence [17]. Five studies were identified in the review, including persons with psychiatric conditions, HIV-positive, drug-involved offenders and people with low back pain. The review included controlled studies and interrupted time series studies.

Several recent publications show that there is a growing interest in MI in vocational rehabilitation [10, 18]. However, it is unclear what evidence exists related to the use of MI to help people with musculoskeletal disorders return to work. We need an updated review of the study field in order to define future research priorities. The review should include both quantitative and qualitative research, as qualitative research can give information about barriers and facilitators to implementing MI for people with musculoskeletal disorders. Thus, the objective of this review was to map all types of empirical research on MI as a method to help people with musculoskeletal disorders return to work. Our research question was: *What research on MI as a method to facilitate return to work for individuals who are on sick leave due to musculoskeletal disorders exists, and what are the results of the research?*

## Method

### Design

We followed the guidelines in the Cochrane Handbook for Systematic Reviews of Interventions [19] and the methodological steps for mapping reviews proposed by Arksey and O’Malley [20] and Levac et al. [21]. The systematic mapping review is reported in accordance with the preferred reporting items for systematic reviews and meta-analyses extension for scoping reviews (PRISMA-ScR) [22].

### Eligibility Criteria

Included studies had to address MI as a method to facilitate return to work for individuals on sick leave or disability pension due to a musculoskeletal disorder. All types of empirical studies were included if they were published after 1983 (the year Miller first described the MI method). Studies were included if at least 50% of the study sample had musculoskeletal disorders, or if results were presented separately for people with these diagnoses. We also wanted to include studies on those giving MI to facilitate return to work for individuals on sick leave with musculoskeletal disorders. Detailed inclusion and exclusion criteria are described in Table 1.

**Table 1:** Eligibility criteria

Participants^a^	*Receivers of MI interventions:* Musculoskeletal disorders main reason for work absence On sick leave (part or full time), receiving work assessment allowance or disability pensions Age group: 18–67 years *Performers of MI interventions*: Person with MI-training using MI to facilitate return to work for participants described above
Concept	MI given as a solo intervention, or in combination with other interventions MI could be given in group sessions, individual meetings or by phone
Context	Any context where MI was being delivered
Study design	All types of empirical studies
Language	English, French, German, Norwegian, Swedish, Danish

*MI* motivational interviewing

^a^Studies were included if 50% of the study population met the inclusion criteria, or if results were reported separately for participants that met the inclusion criteria

### Searches

An information search specialist developed and performed the searches in collaboration with two of the review authors (RB and FA). The search was from 1983 to February 2019, and updated in August 2019. We searched the following electronic databases: MEDLINE (OVID), PsycINFO (OVID), EMBASE (OVID), Cochrane Library (CDSR, CENTRAL) (Wiley), CINAHL (EBSCO), Web of Science Core Collection (SCI-EXPANDED & SSCI) (Clarivate), Sociological Abstracts (ProQuest), Epistemonikos, SveMed + , DARE & HTA (Centre for Reviews and Dissemination). We used different search terms and synonyms for ‘motivational interviewing’, ‘return to work’ and ‘sick leave’. To identify all eligible studies (including studies with mixed populations), we avoided search terms related to musculoskeletal disorders. We did not apply any methodology search filters or language restrictions in the searches. The first author hand searched all issues of the MINT bulletin (the newsletter for MI trainers), searched the journal: motivational interviewing, training, research, implementation, practice (MITRIP) https://www.mitrip.org, the MINT webpage: https://motivationalinterviewing.org and The Norwegian Labour and Welfare Administration (NAV) webpage: https://www.nav.no. We also contacted William Miller and other researchers in the field of MI, to identify ongoing studies or unpublished work. Cited reference searches were performed in Web of science and reference lists of the included papers were hand searched for relevant cited literature by the first author. A detailed description of the search strategy in the databases and other sources is presented in Supplementary material: Appendix I.

### Study Selection

**Fig. 1:**
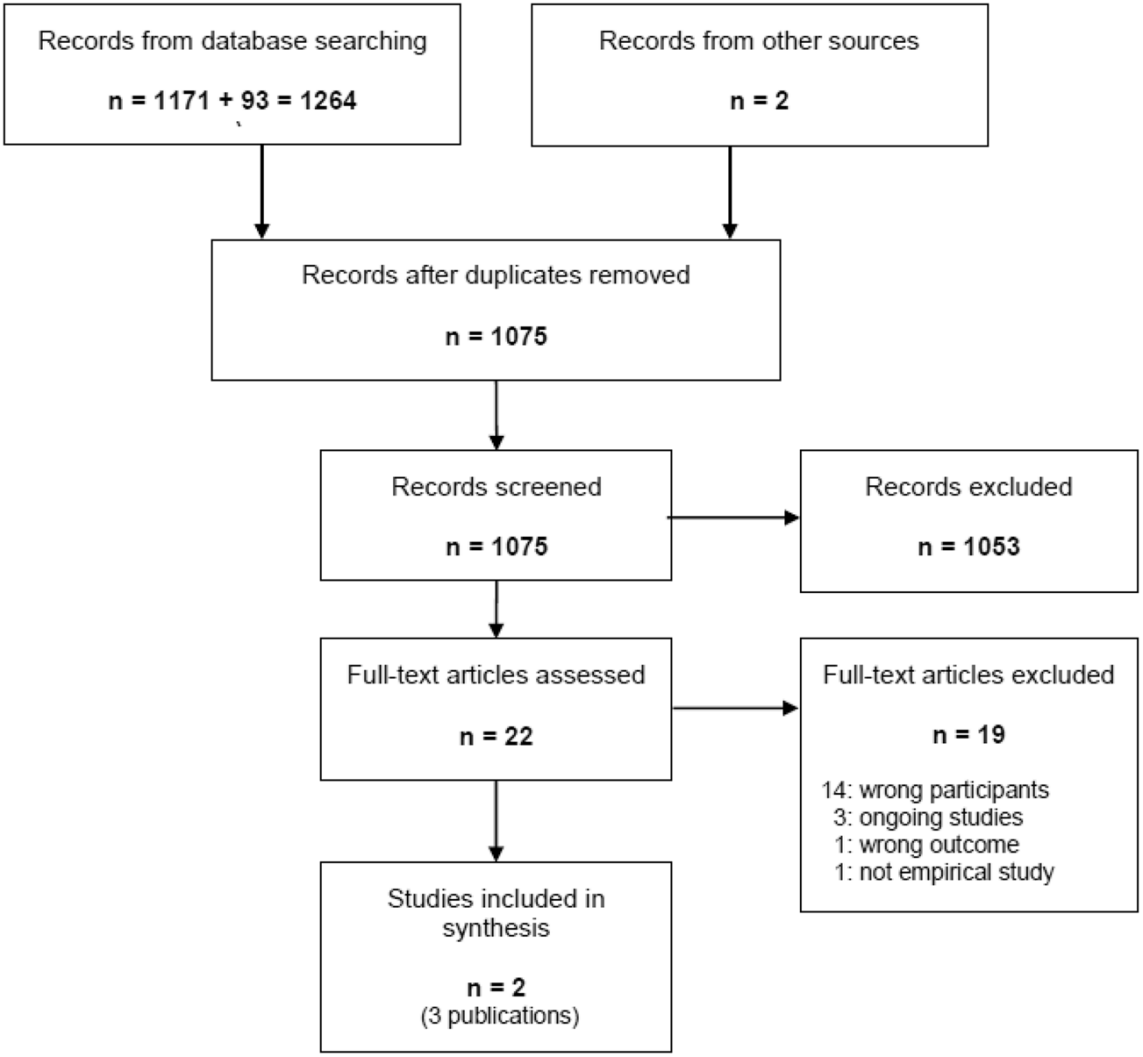
Flow diagram of screening process

The information search specialist imported all the search results from the different databases into the citation management software EndNote (Clarivate Analytics, PA, USA) and removed duplicates. All unique records were imported into the screening tool Rayyan QCRI. Two authors (AT and FA) independently screened abstracts and titles for eligibility, using a pre-designed screening form. Selected studies were screened in full text by two authors separately (IL and FA). At both screening levels, disagreements were resolved by discussion and re-examination of the papers. Figure 1 shows the flow diagram of the study selection process.

### Data Charting and Critical Appraisal

Two authors (MG and FA) independently charted the data from the studies using a predesigned data extraction form. We tested the form and revised it to include more information about study design, participation rate and dropout. The following data were charted from each study: name of first author, year of publication, country, study design, context, study sample/population, participation rate, dropout rate, follow-up period, description of interventions, MI adherence and fidelity, primary and secondary outcomes and results. When data were missing, we contacted study authors to retrieve data.

The included studies were critically appraised by two authors (BEØ and FA) independently, using study specific appraisal checklists [23]. As the only studies meeting the inclusion criteria were RCTs, we used the Cochrane Risk of Bias tool [24]. The judgements of ‘low risk’, ‘high risk’ or ‘unclear risk’ were made for the domains: selection bias, performance bias, detection bias, attrition bias, reporting bias and other bias. We also made a total summary assessment for each study. Papers using data from the same study were appraised as one. Differences in opinion were solved through discussion and re-examination of the studies.

We synthesized the data from the included studies and presented the results narratively and in tables.

## Results

### Search Results

The searches identified 1264 records, of which 1262 were identified through the database searches, one through cited reference searches in Web of science and one through hand searches of the MINT bulletin (Supplementary material: Appendix I). After duplicates were removed, 1075 records remained, and 1053 of these were excluded after screening of titles and abstracts (Fig. 1). Of the 22 publications examined in full-text, 14 were excluded either because less than 50% of the study population had musculoskeletal disorders, or because the proportion of people with these types of disorders in the study sample was not described. Three ongoing studies were excluded due to no published results; one was excluded because it did not have return to work as an aim for the MI intervention, and one because it lacked empirical data (Supplementary material: Appendix II). Three papers from two studies met the inclusion criteria.

### Characteristics of Included Studies

Two of the papers described a Canadian cluster RCT by Gross et al. [25], and Park et al. [26], including 728 claimants, injured at work, with chronic musculoskeletal conditions in different parts of the body. The third paper described a Norwegian RCT by Magnussen et al. including 89 disability pensioners with back pain [27] (Table 2). The two studies included 817 participants, with an average age between 45 and 49 years of whom 60% were male.

**Table 2:** Study characteristics

Author (year) Context	Sample size Population	Design Participation Drop-out	Interventions	MI training and fidelity
Magnussen (2007) No description of setting Norway	N = 89 Disability pensioners with back pain Disability pension > 1 year, mean 8 years 65% women Mean age 49 (SD 5.4) years Range 36–56 years	RCT Random assignment of participants *Experimental:* 45 participants *Comparison:* 44 participants Participation: 21% Dropout: *Experimental:* n = 4 *Comparison:* n = 0 16 did not complete the intervention but were included in the analyses	*Experimental:* Brief vocational intervention programme: 2 × 3 h. group sessions (5–11 in group) 2 h. information about spinal problems + pain mechanisms 1 h. information from social insurance and work office 3 h. MI Medical examination and assessment of work ability by physician and nurse Follow-up from work office for those motivated to RTW *Comparison:* Usual follow-up from social insurance and work office	Not described
Park et al. (2018) Gross et al. (2017) Workers’ compensation rehabilitation facility Canada	N = 728 Claimants with chronic musculoskeletal conditions Mean duration: 234 days 63% men Mean age 45 (SD 12.2) years 73% employed Moderate pain levels Moderate disability	Cluster RCT Random assignment of 12 clinicians: *Experimental:* (367 participants) 4 occupational therapists, 2 exercise therapists *Comparison:* (361 participants) 2 occupational therapists, 4 exercise therapists Participation: 802 claimants assessed, 74 excluded: co-morbid conditions (n = 12) noncompliance/non compensable medical reasons (n = 32) attended program < 5 days (n = 30) No dropout of included clinicians or participants	*Experimental:* Usual care at rehab centre + individual MI sessions number of sessions decided by clinicians (not reported) duration of MI session from 10–50 min *Comparison:* Usual care at rehab centre: Interdisciplinary rehabilitation to improve work abilities (4–6 weeks) Individually tailored functional restoration program including: exercise, graded activity, RTW planning, educational workshops and individual counselling (3–5 days per week, up to 4 h per day)	MI training: 3 full-day sessions + monthly coaching MI fidelity: Completion of MI adherence checklist: MI given to 96 of 367 claimants (26%) range: 4–56%

*RTW* return to work, *MI* motivaiontal interviewing

Both studies investigated the effect of MI on return to work, in combination with other interventions. In the Norwegian study, MI was provided as part of a brief group intervention and compared to usual follow-up from the social insurance and work office. The MI was aimed at helping the participants focus on their strength and capacities, identify barriers for returning to work, and search for ways to succeed in returning to work. In the Canadian study, the comparison group received usual follow-up, consisting of an individually tailored restoration program at a workers’ compensation rehabilitation facility. The experimental group received individual MI conversations in addition to usual follow-up. The clinicians providing the MI were trained to listen for signs of ambivalence and to offer MI to those who were ambivalent about behaviour change. The clinicians decided the number and duration of the MI sessions (Table 2).

In the Norwegian study, a psychologist gave MI during a three-hour group session (information obtained from study author). There was no fidelity or adherence measurements related to the delivery of MI in this study nor any description of the psychologists MI competence. The clinicians giving the intervention in the Canadian study were occupational therapists and exercise therapist who had received 3 days of MI training by qualified MI instructors. They were given monthly coaching sessions during the intervention period. The clinicians completed an MI adherence checklist for each claimant. The checklist included registration of the fundamental processes used in MI and identification of a target behaviour for the MI session. Totally, MI was given to 26% of the claimants in the experimental group (Table 2).

### Critical Appraisal

We rated the Norwegian study as having high risk of bias mainly due to lack of blinding of participants and intervention providers, small sample size and high drop out in the intervention group. The Canadian study was rated as having low risk of bias (Table 3).

**Table 3:** Risk of bias

Bias	Judgement	Support for judgement
*Magnussen et al.* [27], *randomized controlled trial*		
Random sequence generation (selection bias)	Low	Computer-generated random list
Allocation concealment (selection bias)	Low	Concealed random allocation
Blinding of participants and personnel (performance bias)	High	Not possible to blind participants and personnel
Blinding of outcome assessment (detection bias)	Low	Primary outcome 1: reduced disability pensions from register data from National Insurance office
	Unclear	Primary outcome 2: being in a return to work process Self-reported outcome on posted questionnaire, no information about blinding of assessment
Incomplete outcome data (attrition bias)	Unclear	Unclear if data were collected for dropouts
Selective reporting (reporting bias)	Low	No published protocol, report results for all given outcomes
Other bias	High	Only 29/45 completed the intervention Small sample size No description of MI training or fidelity measurement
Summary assessment	High	Plausible bias that seriously weakens confidence in the results because of lack of blinding of participants and personnel, small sample size, low compliance to intervention and unsure fidelity to MI intervention
*Gross et al.* [25]* and Park et al.* [26], *cluster randomized control trial*		
Random sequence generation (selection bias)	Low	Clinicians were randomly allocated to intervention group or control group using a computerized random number generator
Allocation concealment (selection bias)	Unclear	No information given regarding allocation concealment
Blinding of participants and personnel (performance bias)	High	Not possible to blind participants and personnel Participants were unaware of the study and group membership
Blinding of outcome assessment (detection bias)	Low	Data were collected from Workers’ Compensation Board Alberta claims database by blinded outcome assessors
Incomplete outcome data (attrition bias)	Low	Available outcome measures for 100% of sample at time of discharge and during 1-year follow-up
Selective reporting (reporting bias)	Unclear	All primary outcomes reported, no report of secondary outcomes described in protocol Protocol registered retrospectively
Other bias	Low	No other bias identified
Summary assessment	Low	Plausible bias is unlikely to alter the results Not possible to blind participants and personnel, but the main outcome is not likely to be influenced by lack of blinding

### Main Findings from the Studies

The results from the Norwegian study showed no effect on work related outcomes at 1-year follow-up [27]. Only one person in the MI group and two in the comparison group had returned to work at one-year follow-up. There was no statistically significant difference in being in a return to work process between the MI group and the control group (Table 4).

**Table 4:** Main findings

Paper	Results from primary outcomes
Magnussen et al. (2007)	**Reductions in disability pensions**^r^ (range in reductions: 4–42%)
	Experimental group: n = 1 (2%), comparison group: n = 2 (4.5%), non-attendees: n = 4 (1%), *ns*
	**In RTW process at one year follow up**^s^
	Experimental group: n = 10 (22%), comparison group: n = 5 (11%) RR 1.96 (95% CI 0.73–5.26) Power calculations: power of difference: 19%. Absolute risk reduction: 11. Number needed to treat: 9.2 (95% CI 3.4, Inf)
Park et al. (2018)	**RTW at discharge**^r^
	Claimants unemployed at baseline:
	Experimental group: 21.6% RTW, comparison group: 9.5% RTW (p = 0.03) MI adherent clinicians^a^: 33.3% RTW, non-adherent clinicians: 18.0% RTW, comparison group: 9.5% RTW (p < 0.01) Multivariable analysis adjusting for: age, sex, annual salary, marital status, pain intensity, disability and therapist cluster: OR for RTW in experimental group compared to comparison group: 2.64 (95% CI 0.69–10.14)
	Claimants employed at baseline:
	Experimental group: 97.1% RTW, comparison group: 94.1% RTW, *ns* MI adherent clinicians^a^: 100% RTW, non-adherent clinicians: 96.3% RTW, comparison group: 94.1% RTW (p = 0.03) Multivariable analysis adjusting for: age, sex, annual salary, marital status, pain intensity, disability and therapist cluster: OR for RTW in experimental group compared to comparison group: 2.50 (95% CI 0.68–9.14)
Gross et al. (2017)	**Number of days receiving wage replacement benefits in the follow-up year**^r^
	Claimants unemployed at baseline:
	*Partial temporary disability benefits*: experimental group: 8.2 days (SD 28.1), comparison group: 0.2 days (SD 1.5) (p < 0.001) Multivariable analysis adjusted for age, sex, previous claims, preinjury annual salary, self-rated disability and pain intensity: B 0.15 (95% CI 0.01–0.30) Percent of clients receiving partial temporary disability benefits: MI adherent clinicians^a^: 18.7%, non-adherent clinicians: 5.2%, comparison: 0.2% (p = 0.001)
	Claimants employed at baseline:
	*Job search allowance*: experimental group 3.1 days (SD 13.6), comparison group: 1 day (SD 7.9) (p = 0.01)
	**Recurrence of wage replacement benefits in the follow up yea**r^r^
	Claimants employed at baseline:
	*Recurrence of any type of wage replacement benefits*: experimental group: 4.5% recurrence, comparison group: 9.1% recurrence (p = 0.04) Multivariate analysis adjusted for age, sex, previous claims, preinjury annual salary, self-rated disability and pain intensity: OR for recurrence of wage replacement benefits in comparison group compared to experimental group: 2.01 (95% CI 0.96–4.21) MI adherent clinicians^a^: 2.9% recurrence, non-adherent clinicians: 5.2% recurrence, comparison group: 9.1% recurrence (p = 0.02)
	*Recurrence of partial temporary disability benefits*: experimental group 2.9% recurrence, comparison group: 7.7% recurrence (p = 0.02) Multivariable analysis adjusted for age, sex, previous claims, preinjury annual salary, self-rated disability and pain intensity: OR for recurrence of partial temporary disability benefits in comparison group compared to experimental group 2.69 (95% CI 1.12–6.46) MI adherent clinicians^a^ 0% recurrence, non-adherent clinicians: 4.0% recurrence, comparison group: 7.7% recurrence (p = 0.002)

^r^ = registry data, ^s^ = self-report, *RTW* return to work, *ns* not statistically significant difference, *RR* relative risk

^a^Clinicians documented MI use on adherence checklists

Results from the Canadian study showed that 12.1% more of the claimants, who were unemployed at baseline, had returned to work at discharge in the MI group compared to those receiving usual care only (p = 0.03). There were no statistically significant difference in return to work between the MI group and the comparison group among those employed at baseline. At one-year follow-up, claimants in the MI group who were unemployed at baseline received 8 days more of partial temporary disability benefits than the comparison group (p = 0.02), indicating that more claimants in the MI group had returned to modified work duties. The claimants in the MI group, who were employed at baseline, had 4.6% less recurrence of any type of benefits than the comparison group (p = 0.04) [25]. The effects in the Canadian study were significantly higher among the claimants of the MI adherent clinicians compared to the non-adherent clinicians. All the workers who were employed at baseline and treated by the MI adherent clinicians had returned to work at discharge. Among the claimants who were unemployed at baseline, three times as many of the clients who were treated by the MI adherent clinicians returned to work, compared to those receiving usual follow-up (Table 4).

## Discussion

This is the first systematic mapping review of the evidence of MI to facilitate return to work for people with musculoskeletal disorders. We identified only three published papers from two RCTs. The RCTs had inconsistent results regarding the effect of MI on return to work for people with chronic musculoskeletal disorder. This is in line with previous systematic reviews which have shown that there are few studies on MI for people with chronic pain [13] and musculoskeletal disorders [12]. A meta-review from 2018 found moderate quality of evidence of the effectiveness of MI in promoting physical activity for people with chronic health conditions [28], while a systematic review from 2016 found small to moderate short-time effects of MI on treatment adherence and pain reduction for people with chronic pain [13]. Currently, there is limited evidence for the use of MI for people with musculoskeletal disorders due to the small amount and varying quality of studies [12].

There were several methodological differences across the two RCTs included in this mapping review. The Norwegian study included disability pensioners who had been away from work for an average of 8 years. In order to return to work after several years of absence, the disability pensioners might have to retrain and spend time searching for jobs [29]. At the one-year follow-up, twice as many in the experimental group reported being in a return to work process [27]. Some of these participants may have returned to work if the study follow-up period was longer. In addition, the study had a small sample size and only 64% in the intervention group completed the intervention. The study also lacked a description of the MI competence of the psychologist providing the intervention. For MI to be effective, the clinician should build a good working alliance [11] and elicit and amplify the persons change talk [30]. This may be challenging to accomplish during a single group session of MI even for a trained psychologist. Finally, MI was only one of several components of the brief group intervention, making it impossible to separate the effects of MI from the rest of the intervention.

In the Canadian study 73% of the study population were still employed at baseline and the mean time away from work for all the participants was less than one year. Among the claimants who were employed at baseline, there was a very high return to work rate both in the MI group and in the comparison group. This could have resulted in a ceiling effect, making it hard to detect any benefit of the MI intervention. In addition, only one fourth of the claimants in the experimental group received MI, which could have reduced the effectiveness of the intervention. Subgroup analyses showed that the effects on return to work in the MI group were highest among the claimants of the most MI adherent clinicians. This was the case both at discharge and at one-year follow-up among all the workers [25, 26]. The results suggest that the MI intervention might have been more effective if adherence had been higher among the clinicians. In the Canadian study the experimental group received MI in addition to usual follow-up, while the comparison group received usual follow-up only. We can therefore assume that MI contributed to the larger effect in the intervention group.

Surprisingly, the searches did not identify qualitative studies investigating how people with musculoskeletal disorders experience receiving MI to help them return to work, or how people who deliverer MI to people with musculoskeletal disorders experience the intervention. There are, however, several qualitative studies from the Swedish Dirigo project. In this project insurance officials were trained in MI to facilitate return to work for people on sick leave. Although these studies included people with all types of diagnoses, the results may be relevant for people with musculoskeletal disorders. Andersen and colleagues interviewed fourteen people on long term sick leave (mainly with mental disorders) about their experiences from the Dirigo project. The informants had positive experiences of MI because they felt the method helped them get to know themselves better, and become aware of opportunities for work and studies. They also felt that the insurance officials were making an effort to get to know them and their situation [31]. Two studies by Stahl and colleagues, from the same project, interviewed insurance officials in charge of following up people on sick leave [32, 33] and a study by Secker and Margrove investigated employment support workers experiences of MI [34]. These studies showed that the professionals were positive to MI and found it helpful in their work. However, they emphasized the need for support and ongoing assessment of MI skills in order to become confident in practicing MI and able to use the method in their work with clients. Lack of training, confidence and support in performing MI are common challenges reported by practitioners [33, 35].

Despite comprehensive literature searches by an experienced search specialist, one limitation of this review is that we might have missed grey literature. A strength of this review is its focus on musculoskeletal disorder as they are the main cause of disability. Another strength is the broad search strategy and inclusion criteria making it possible to include all relevant studies in the mapping review.

The current review has shown that there is a lack of research on MI for people with musculoskeletal disorders. In order to assess the effectiveness of MI on return to work for people with musculoskeletal disorders, we need more high-quality intervention studies. The studies should include adequate MI training for the persons delivering the intervention and assessment of their MI skills [13]. There appears to be increasing research interest in the use of MI in vocational rehabilitation. This review identified three ongoing trials including both qualitative and quantitative studies, and 14 publications on the use of MI in vocational rehabilitation for mixed populations of people with different conditions.

Although MI has been recommended as a method in vocational rehabilitation [10], the recommendations seem to be based primarily on theoretical papers describing the compatibility between MI, and aims and values in vocational rehabilitation [9, 18, 35, 36]. The current review has revealed a huge research gap on the use of MI to facilitate return to work for people with musculoskeletal disorders. Only two efficacy studies of variable methodological quality, with conflicting results were available. Hence, more studies should be conducted before MI is implemented as a method to increase return to work for patients on sick leave with musculoskeletal disorders.

## Electronic supplementary material

Below is the link to the electronic supplementary material.

Supplementary file1 (DOCX 57 kb)
