# Supplementary material for: Motivational Interviewing and Return to Work for People with Musculoskeletal Disorders: A Systematic Mapping Review
Source: J Occup Rehabil. 2020 Apr 30;31(1):63–71. doi: 10.1007/s10926-020-09892-0 (PMC7954732; doi:10.1007/s10926-020-09892-0)
Supplement: Supplementary file 1 — Supplementary file1 (DOCX 57 kb) [file 10926_2020_9892_MOESM1_ESM.docx]

## Appendix I: Search strategy

INITIAL DATABASE SEARCHES

**Database: Ovid MEDLINE(R) and Epub Ahead of Print, In-Process & Other Non-Indexed Citations and Daily 1946 to January 28, 2019**

**Date: 04.02.19**

**Records: 555**

1 return to work/ 1902

2 employment, supported/ 1129

3 rehabilitation, vocational/ 9272

4 sick leave/ 5383

5 absenteeism/ 8632

6 (sickleave* or sicklist* or (sick* adj (absen* or allowance* or benefit* or certifi* or day* or insurance* or leave* or list*)) or (medical adj (absen* or leave*)) or certified absen* or (illness adj2 (day* or absen*)) or (work adj2 absen*) or re-employ* or reemploy* or "back to work" or ((work* or job or employment) adj2 (return* or re-ent* or reent*)) or ((vocational or occupational) adj1 (rehab* or reintegration* or re-integration*)) or supported employment or (disability adj (absen* or allowance* or benefit* or insurance* or leave* or pension*)) or (work* adj (compensation* or resumption*))).ti,ab,kf. 32519

7 or/1-6 47021

8 motivational interview/ 1423

9 motivational interview*.ti,ab,kf. 3710

10 motivational enhancement.ti,ab,kf. 491

11 motivation/ 61923

12 or/8-11 65053

13 7 and 12 691

14 exp animals/ 22060973

15 humans/ 17519806

16 14 not (14 and 15) 4541167

17 (news or editorial or comment).pt. 1275551

18 13 not (16 or 17) 683

19 limit 18 to yr="1983-current" 556

20 remove duplicates from 19 555

**Database: PsycINFO 1806 to January Week 3 2019 (OVID)**

**Date: 04.02.2019**

**Records: 146**

1 reemployment/ 1325

2 supported employment/ 1284

3 vocational rehabilitation/ 5831

4 employee leave benefits/ 1012

5 (sickleave* or sicklist* or (sick* adj (absen* or allowance* or benefit* or certifi* or day* or insurance* or leave* or list*)) or (medical adj (absen* or leave*)) or certified absen* or (illness adj2 (day* or absen*)) or (work adj2 absen*) or re-employ* or reemploy* or "back to work" or ((work* or job or employment) adj2 (return* or re-ent* or reent*)) or ((vocational or occupational) adj1 (rehab* or reintegration* or re-integration*)) or supported employment or (disability adj (absen* or allowance* or benefit* or insurance* or leave* or pension*)) or (work* adj (compensation* or resumption*))).ti,ab,id. 14253

6 or/1-5 17196

7 motivational interviewing/ 2233

8 motivational interview*.ti,ab,id. 3449

9 motivational enhancement.ti,ab,id. 649

10 motivation/ 49511

11 or/7-10 52905

12 6 and 11 162

13 limit 12 to yr="1983-Current" 146

14 remove duplicates from 13 146

**Database: Embase 1974 to 2019 January 29 (OVID)**

**Date: 04.02.2019**

**Records: 54**

1 *return to work/ 1235

2 *work resumption/ 1044

3 *vocational rehabilitation/ 4350

4 *medical leave/ 1793

5 *absenteeism/ 4939

6 (sickleave* or sicklist* or (sick* adj (absen* or allowance* or benefit* or certifi* or day* or insurance* or leave* or list*)) or (medical adj (absen* or leave*)) or certified absen* or (illness adj2 (day* or absen*)) or (work adj2 absen*) or re-employ* or reemploy* or "back to work" or ((work* or job or employment) adj2 (return* or re-ent* or reent*)) or ((vocational or occupational) adj1 (rehab* or reintegration* or re-integration*)) or supported employment or (disability adj (absen* or allowance* or benefit* or insurance* or leave* or pension*)) or (work* adj (compensation* or resumption*))).ti,ab,kw. 39992

7 or/1-6 45353

8 motivational interviewing/ 3938

9 motivational interview*.ti,ab,kw. 5445

10 motivational enhancement.ti,ab,kw. 687

11 *motivation/ 24713

12 or/8-11 31150

13 7 and 12 173

14 exp animals/ or exp invertebrate/ or animal experiment/ or animal model/ or animal tissue/ or animal cell/ or nonhuman/ 25425730

15 human/ or normal human/ or human cell/ 19370012

16 14 not (14 and 15) 6108565

17 (news or editorial or comment).pt. 595619

18 13 not (16 or 17) 173

19 limit 18 to yr="1983-current" 153

20 limit 19 to embase 55

21 remove duplicates from 20 54

**Database: Cochrane Library (CDSR, CENTRAL & Cochrane Protocols)**

**Date: 04.02.2019**

**Records: 77**

#1 [mh ^"return to work"] 189

#2 [mh ^"employment, supported"] 118

#3 [mh ^"rehabilitation, vocational"] 366

#4 [mh ^"sick leave"] 522

#5 [mh ^absenteeism] 465

#6 (sickleave* or sicklist* or (sick* NEXT (absen* or allowance* or benefit* or certifi* or day* or insurance* or leave* or list*)) or (medical NEXT (absen* or leave*)) or certified NEXT absen* or (illness NEAR/2 (day* or absen*)) or (work NEAR/2 absen*) or re-employ* or reemploy* or "back to work" or ((work* or job or employment) NEAR/2 (return* or re-ent* or reent*)) or ((vocational or occupational) NEAR/1 (rehab* or reintegration* or re-integration*)) or "supported employment" or (disability NEXT (absen* or allowance* or benefit* or insurance* or leave* or pension*)) or (work* NEXT (compensation* or resumption*))):ti,ab 3575

#7 (2-#6-#6) 4098

#8 [mh ^"motivational interview"] 637

#9 (motivational NEXT interview*):ti,ab 2223

#10 "motivational enhancement":ti,ab 390

#11 [mh ^motivation] 4267

#12 {or #8-#11 6437

#13 #7 and #12 with Cochrane Library publication date Between Jan 1983 and Feb 2019, in Cochrane Reviews 2

#14 #7 and #12 with Publication Year from 1983 to 2019, in Trials 71

#15 (sickleave* or sicklist* or (sick* NEXT (absen* or allowance* or benefit* or certifi* or day* or insurance* or leave* or list*)) or (medical NEXT (absen* or leave*)) or certified NEXT absen* or (illness NEAR/2 (day* or absen*)) or (work NEAR/2 absen*) or re-employ* or reemploy* or "back to work" or ((work* or job or employment) NEAR/2 (return* or re-ent* or reent*)) or ((vocational or occupational) NEAR/1 (rehab* or reintegration* or re-integration*)) or "supported employment" or (disability NEXT (absen* or allowance* or benefit* or insurance* or leave* or pension*)) or (work* NEXT (compensation* or resumption*))) 4768

#16 (motivational NEXT interview* or "motivational enhancement") 3184

#17 #15 and #16 in Cochrane Protocols 4

#18 #13 or #14 or #17 77

**Database: CINAHL (EBSCO)**

**Date: 04.02.2019**

**Records: 209**

S1 (MH Job Re-Entry) (5,453)

S2 (MH Rehabilitation, Vocational) (5,281)

S3 (MH "Employment, Supported") (949)

S4 (MH "Sick Leave") (4,249)

S5 (MH "Absenteeism") (4,013)

S6 TI ( (sickleave* or sicklist* or (sick* W0 (absen* or allowance* or benefit* or certifi* or day* or insurance* or leave* or list*)) or (medical W0 (absen* or leave*)) or certified W0 absen* or (illness N1 (day* or absen*)) or (work N1 absen*) or re-employ* or reemploy* or "back to work" or ((work* or job or employment) N1 (return* or re-ent* or reent*)) or ((vocational or occupational) N0 (rehab* or reintegration* or re-integration*)) or "supported employment" or (disability W0 (absen* or allowance* or benefit* or insurance* or leave* or pension*)) or (work* W0 (compensation* or resumption*))) ) OR AB ( (sickleave* or sicklist* or (sick* W0 (absen* or allowance* or benefit* or certifi* or day* or insurance* or leave* or list*)) or (medical W0 (absen* or leave*)) or certified W0 absen* or (illness N1 (day* or absen*)) or (work N1 absen*) or re-employ* or reemploy* or "back to work" or ((work* or job or employment) N1 (return* or re-ent* or reent*)) or ((vocational or occupational) N0 (rehab* or reintegration* or re-integration*)) or "supported employment" or (disability W0 (absen* or allowance* or benefit* or insurance* or leave* or pension*)) or (work* W0 (compensation* or resumption*))) )

(10,018)

S7 S1 OR S2 OR S3 OR S4 OR S5 OR S6 (22,132)

S8 (MH "Motivational Interviewing") (2,674)

S9 TI ( motivational W0 interview* or "motivational enhancement" ) OR AB ( motivational W0 interview* or "motivational enhancement" ) (2,832)

S10 (MH "Motivation") (31,513)

S11 S8 OR S9 OR S10 (34,865)

S12 S7 AND S11 [Limiters - Exclude MEDLINE records; Published Date: 19830101-20190204] 209

**Database: Web of science (Clarivate)**

**Date: 04.02**

**Records: 21**

# 1 TOPIC: ((sickleave* OR sicklist* OR "sickness ansence" OR "sickness allowance" OR "sickness allowances" OR "sickness benefit" OR "sickness benefits" OR "sickness certification" OR "sickness certifications" OR "sick day" OR "sick days" OR "sickness insurance" OR "sickness insurances" OR "sick leave" OR "sick leaves" OR "sick list" OR "sick lists" OR "sick listed" OR "sick listing" OR "sick listings" OR "medical ansence" OR "medical ansences" OR "medical leave" OR "medical leaves" OR "certified absence" OR "certified ansences" OR "illness day" OR "illness days" OR "illness ansence" OR "illness ansences" OR "work ansence" OR "work ansences" OR re-employ* OR reemploy* OR "return to work" OR "return-to-work" OR "returning to work" OR "back to work" OR "back-to-work" OR "vocational rehabilitation" OR "vocational reintegration" OR "vocational re-integration" OR "occupational rehabilitation" OR "occupational re-integration" OR "occupational reintegration" OR "supported employment" OR "disability ansence" OR "disability ansences" OR "disability allowance" OR "disability allowances" OR "disability benefit" OR "disability benefits" OR "disability insurance" OR "disability insurances" OR "disability leave" OR "disability leaves" OR "disability pension" OR "disability pensions" OR "workers compensation" OR "workers’ compensation" OR "work resumption" OR "work resumptions")) 21,798

# 2 TOPIC: (("motivational interviewing" OR "motivational interview" OR "motivational interviews" OR "motivational enhancement")) 4,354

# 3 #2 AND #1 [Indexes=SCI-EXPANDED, SSCI Timespan=1987-2019] 21

**Database: Sociological Abstracts (ProQuest)**

**Date: 04.02.2019**

**Records: 62**

(MAINSUBJECT.EXACT("Vocational Rehabilitation") OR (sickleave* OR sicklist* OR (sick* PRE/0 (absen* OR allowance* OR benefit* OR certifi* OR day* OR insurance* OR leave* OR list*)) OR (medical PRE/0 (absen* OR leave*)) OR certified PRE/0 absen* OR (illness NEAR/1 (day* OR absen*)) OR (wORk NEAR/1 absen*) OR re-employ* OR reemploy* OR "back to wORk" OR ((wORk* OR job OR employment) NEAR/1 (return* OR re-ent* OR reent*)) OR ((vocational OR occupational) NEAR/0 (rehab* OR reintegration* OR re-integration*)) OR "suppORted employment" OR (disability PRE/0 (absen* OR allowance* OR benefit* OR insurance* OR leave* OR pension*)) OR (wORk* PRE/0 (compensation* OR resumption*)))) AND (MAINSUBJECT.EXACT("Motivation") OR Ti,AB,SU(motivational PRE/0 interview* OR "motivational enhancement")) [Limit applied: 1983-01-01 – 2019-01-31] 62

**Database: SveMed+**

**Date: 04.02.2019**

**Records: 44**

1 noexp:"Motivational Interviewing" 37

2 ("motivational interviewing" OR "motivational interview" OR "motivational interviews" OR "motivational enhancement") 44

3 #1 OR #2 44

**Database: DARE (The Database of Abstracts of Reviews of Effects) & HTA (Health Technology Assessment)**

**Date: 31.01.2019**

**Records: 1**

1 MeSH DESCRIPTOR return to work IN DARE,HTA 15

2 MeSH DESCRIPTOR employment, supported IN DARE,HTA 8

3 MeSH DESCRIPTOR rehabilitation, vocational IN DARE,HTA 28

4 MeSH DESCRIPTOR sick leave IN DARE,HTA 27

5 MeSH DESCRIPTOR absenteeism IN DARE,HTA 20

6 ((sickleave* or sicklist* or (sick* adj (absen* or allowance* or benefit* or certifi* or day* or insurance* or leave* or list*)) or (medical adj (absen* or leave*)) or certified absen* or (illness adj2 (day* or absen*)) or (work adj2 absen*) or re-employ* or reemploy* or "back to work" or ((work* or job or employment) adj2 (return* or re-ent* or reent*)) or ((vocational or occupational) adj1 (rehab* or reintegration* or re-integration*)) or supported employment or (disability adj (absen* or allowance* or benefit* or insurance* or leave* or pension*)) or (work* adj (compensation* or resumption*)))) IN DARE, HTA 242

7 #1 OR #2 OR #3 OR #4 OR #5 OR #6 269

8 MeSH DESCRIPTOR Motivational Interviewing IN DARE,HTA 27

9 (motivational interview*) IN DARE, HTA 111

10 (motivational enhancement) IN DARE, HTA 9

11 MeSH DESCRIPTOR Motivation IN DARE,HTA 121

12 #8 OR #9 OR #10 OR #11 210

13 #7 AND #12 1

**Database: Epistemonikos**

**Date: 04.02.2019**

**Records: 2**

(sickleave* OR sicklist* OR "sickness absence" OR "sickness allowance" OR "sickness allowances" OR "sickness benefit" OR "sickness benefits" OR "sickness certification" OR "sickness certifications" OR "sick day" OR "sick days" OR "sickness insurance" OR "sickness insurances" OR "sick leave" OR "sick leaves" OR "sick list" OR "sick lists" OR "sick listed" OR "sick listing" OR "sick listings" OR "medical absence" OR "medical absences" OR "medical leave" OR "medical leaves" OR "certified absence" OR "certified absences" OR "illness day" OR "illness days" OR "illness absence" OR "illness absences" OR "work absence" OR "work absences" OR re-employ* OR reemploy* OR "return to work" OR "return-to-work" OR "returning to work" OR "back to work" OR "back-to-work" OR "vocational rehabilitation" OR "vocational reintegration" OR "vocational re-integration" OR "occupational rehabilitation" OR "occupational re-integration" OR "occupational reintegration" OR "supported employment" OR "disability absence" OR "disability absences" OR "disability allowance" OR "disability allowances" OR "disability benefit" OR "disability benefits" OR "disability insurance" OR "disability insurances" OR "disability leave" OR "disability leaves" OR "disability pension" OR "disability pensions" OR "workers compensation" OR "workers’ compensation" OR "work resumption" OR "work resumptions") AND ("motivational interviewing" OR "motivational interview" OR "motivational interviews" OR "motivational enhancement")

UPDATED DATABASE SEARCHES

**Database: Ovid MEDLINE(R) and Epub Ahead of Print, In-Process & Other Non-Indexed Citations and Daily 1946 to August 13, 2019**

**Date: 14.08.2019**

**Records: 568**

# Searches Results

1 return to work/ 2111

2 employment, supported/ 1147

3 rehabilitation, vocational/ 9319

4 sick leave/ 5530

5 absenteeism/ 8786

6 (sickleave* or sicklist* or (sick* adj (absen* or allowance* or benefit* or certifi* or day* or insurance* or leave* or list*)) or (medical adj (absen* or leave*)) or certified absen* or (illness adj2 (day* or absen*)) or (work adj2 absen*) or re-employ* or reemploy* or "back to work" or ((work* or job or employment) adj2 (return* or re-ent* or reent*)) or ((vocational or occupational) adj1 (rehab* or reintegration* or re-integration*)) or supported employment or (disability adj (absen* or allowance* or benefit* or insurance* or leave* or pension*)) or (work* adj (compensation* or resumption*))).ti,ab,kf. 33318

7 or/1-6 48004

8 motivational interview/ 1561

9 motivational interview*.ti,ab,kf. 3910

10 motivational enhancement.ti,ab,kf. 505

11 motivation/ 63754

12 or/8-11 67095

13 7 and 12 703

14 exp animals/ 22518938

15 humans/ 17910345

16 14 not (14 and 15) 4608593

17 (news or editorial or comment).pt. 1322044

18 13 not (16 or 17) 695

19 limit 18 to yr="1983-current" 568

**Database(s): Embase 1974 to 2019 August 13**

**Date: 14.08.2019**

**Records: 57**

# Searches Results

1 *return to work/ 1433

2 *work resumption/ 1049

3 *vocational rehabilitation/ 4405

4 *medical leave/ 1853

5 *absenteeism/ 5057

6 (sickleave* or sicklist* or (sick* adj (absen* or allowance* or benefit* or certifi* or day* or insurance* or leave* or list*)) or (medical adj (absen* or leave*)) or certified absen* or (illness adj2 (day* or absen*)) or (work adj2 absen*) or re-employ* or reemploy* or "back to work" or ((work* or job or employment) adj2 (return* or re-ent* or reent*)) or ((vocational or occupational) adj1 (rehab* or reintegration* or re-integration*)) or supported employment or (disability adj (absen* or allowance* or benefit* or insurance* or leave* or pension*)) or (work* adj (compensation* or resumption*))).ti,ab,kw. 41713

7 or/1-6 47130

8 motivational interviewing/ 4357

9 motivational interview*.ti,ab,kw. 5854

10 motivational enhancement.ti,ab,kw. 709

11 *motivation/ 25720

12 or/8-11 32663

13 7 and 12 181

14 exp animals/ or exp invertebrate/ or animal experiment/ or animal model/ or animal tissue/ or animal cell/ or nonhuman/ 26317806

15 human/ or normal human/ or human cell/ 20098939

16 14 not (14 and 15) 6277136

17 (news or editorial or comment).pt. 627658

18 13 not (16 or 17) 181

19 limit 18 to yr="1983-current" 161

20 limit 19 to embase 58

21 remove duplicates from 20 57

**Database: PsycINFO 1806 to August Week 1 2019**

**Date: 14.08.2019**

**Records: 150**

# Searches Results

1 reemployment/ 1380

2 supported employment/ 1333

3 vocational rehabilitation/ 5891

4 employee leave benefits/ 1039

5 (sickleave* or sicklist* or (sick* adj (absen* or allowance* or benefit* or certifi* or day* or insurance* or leave* or list*)) or (medical adj (absen* or leave*)) or certified absen* or (illness adj2 (day* or absen*)) or (work adj2 absen*) or re-employ* or reemploy* or "back to work" or ((work* or job or employment) adj2 (return* or re-ent* or reent*)) or ((vocational or occupational) adj1 (rehab* or reintegration* or re-integration*)) or supported employment or (disability adj (absen* or allowance* or benefit* or insurance* or leave* or pension*)) or (work* adj (compensation* or resumption*))).ti,ab,id. 14502

6 or/1-5 17470

7 motivational interviewing/ 2294

8 motivational interview*.ti,ab,id. 3551

9 motivational enhancement.ti,ab,id. 658

10 motivation/ 50429

11 or/7-10 53935

12 6 and 11 166

13 limit 12 to yr="1983-Current" 150

14 remove duplicates from 13 150

**Database: CINAHL (EBSCO)**

**Date: 14.08.2019**

**Records: 217**

S1 (MH Job Re-Entry) (5,453)

S2 (MH Rehabilitation, Vocational) (5,281)

S3 (MH "Employment, Supported") (949)

S4 (MH "Sick Leave") (4,249)

S5 (MH "Absenteeism") (4,013)

S6 TI ( (sickleave* or sicklist* or (sick* W0 (absen* or allowance* or benefit* or certifi* or day* or insurance* or leave* or list*)) or (medical W0 (absen* or leave*)) or certified W0 absen* or (illness N1 (day* or absen*)) or (work N1 absen*) or re-employ* or reemploy* or "back to work" or ((work* or job or employment) N1 (return* or re-ent* or reent*)) or ((vocational or occupational) N0 (rehab* or reintegration* or re-integration*)) or "supported employment" or (disability W0 (absen* or allowance* or benefit* or insurance* or leave* or pension*)) or (work* W0 (compensation* or resumption*))) ) OR AB ( (sickleave* or sicklist* or (sick* W0 (absen* or allowance* or benefit* or certifi* or day* or insurance* or leave* or list*)) or (medical W0 (absen* or leave*)) or certified W0 absen* or (illness N1 (day* or absen*)) or (work N1 absen*) or re-employ* or reemploy* or "back to work" or ((work* or job or employment) N1 (return* or re-ent* or reent*)) or ((vocational or occupational) N0 (rehab* or reintegration* or re-integration*)) or "supported employment" or (disability W0 (absen* or allowance* or benefit* or insurance* or leave* or pension*)) or (work* W0 (compensation* or resumption*))) )

(10,018)

S7 S1 OR S2 OR S3 OR S4 OR S5 OR S6 (22,132)

S8 (MH "Motivational Interviewing") (2,674)

S9 TI ( motivational W0 interview* or "motivational enhancement" ) OR AB ( motivational W0 interview* or "motivational enhancement" ) (2,832)

S10 (MH "Motivation") (31,513)

S11 S8 OR S9 OR S10 (34,865)

S12 S7 AND S11 [Limiters - Exclude MEDLINE records; Published Date: 19830101-20190131] 217

**Database: Cochrane Library (CDSR, CENTRAL & Cochrane Protocols)**

**Date: 14.08.2019**

**Records: 87**

ID Search Hits

#1 [mh ^"return to work"] 202

#2 [mh ^"employment, supported"] 122

#3 [mh ^"rehabilitation, vocational"] 373

#4 [mh ^"sick leave"] 540

#5 [mh ^absenteeism] 477

#6 (sickleave* or sicklist* or (sick* NEXT (absen* or allowance* or benefit* or certifi* or day* or insurance* or leave* or list*)) or (medical NEXT (absen* or leave*)) or certified NEXT absen* or (illness NEAR/2 (day* or absen*)) or (work NEAR/2 absen*) or re-employ* or reemploy* or "back to work" or ((work* or job or employment) NEAR/2 (return* or re-ent* or reent*)) or ((vocational or occupational) NEAR/1 (rehab* or reintegration* or re-integration*)) or "supported employment" or (disability NEXT (absen* or allowance* or benefit* or insurance* or leave* or pension*)) or (work* NEXT (compensation* or resumption*))):ti,ab 4859

#7 {or #1-#6} 5387

#8 [mh ^"motivational interview"] 695

#9 (motivational NEXT interview*):ti,ab,kw 3298

#10 "motivational enhancement":ti,ab 446

#11 [mh ^motivation] 4442

#12 (39-#11-#11) 7518

#13 #7 and #12 with Cochrane Library publication date Between Jan 1983 and Feb 2019, in Cochrane Reviews 2

#14 #7 and #12 with Publication Year from 1983 to 2019, in Trials 81

#15 (sickleave* or sicklist* or (sick* NEXT (absen* or allowance* or benefit* or certifi* or day* or insurance* or leave* or list*)) or (medical NEXT (absen* or leave*)) or certified NEXT absen* or (illness NEAR/2 (day* or absen*)) or (work NEAR/2 absen*) or re-employ* or reemploy* or "back to work" or ((work* or job or employment) NEAR/2 (return* or re-ent* or reent*)) or ((vocational or occupational) NEAR/1 (rehab* or reintegration* or re-integration*)) or "supported employment" or (disability NEXT (absen* or allowance* or benefit* or insurance* or leave* or pension*)) or (work* NEXT (compensation* or resumption*))) 5939

#16 (motivational NEXT interview* or "motivational enhancement") 3853

#17 #15 and #16 in Cochrane Protocols 4

#18 #13 or #14 or #17 87

**Database: Web of Science (Clarivate)**

**Date: 14.08.2019**

**Records: 23**

# 1 TOPIC: ((sickleave* OR sicklist* OR "sickness ansence" OR "sickness allowance" OR "sickness allowances" OR "sickness benefit" OR "sickness benefits" OR "sickness certification" OR "sickness certifications" OR "sick day" OR "sick days" OR "sickness insurance" OR "sickness insurances" OR "sick leave" OR "sick leaves" OR "sick list" OR "sick lists" OR "sick listed" OR "sick listing" OR "sick listings" OR "medical ansence" OR "medical ansences" OR "medical leave" OR "medical leaves" OR "certified absence" OR "certified ansences" OR "illness day" OR "illness days" OR "illness ansence" OR "illness ansences" OR "work ansence" OR "work ansences" OR re-employ* OR reemploy* OR "return to work" OR "return-to-work" OR "returning to work" OR "back to work" OR "back-to-work" OR "vocational rehabilitation" OR "vocational reintegration" OR "vocational re-integration" OR "occupational rehabilitation" OR "occupational re-integration" OR "occupational reintegration" OR "supported employment" OR "disability ansence" OR "disability ansences" OR "disability allowance" OR "disability allowances" OR "disability benefit" OR "disability benefits" OR "disability insurance" OR "disability insurances" OR "disability leave" OR "disability leaves" OR "disability pension" OR "disability pensions" OR "workers compensation" OR "workers’ compensation" OR "work resumption" OR "work resumptions")) 21,798

# 2 TOPIC: (("motivational interviewing" OR "motivational interview" OR "motivational interviews" OR "motivational enhancement")) 4,354

# 3 #2 AND #1 [Indexes=SCI-EXPANDED, SSCI Timespan=1987-2019] 23

**Database: Sociological Abstracts & Social Services Abstracts**

**Date: 14.08.2019**

**Records: 118**

(MAINSUBJECT.EXACT("Vocational Rehabilitation") OR (sickleave* OR sicklist* OR (sick* PRE/0 (absen* OR allowance* OR benefit* OR certifi* OR day* OR insurance* OR leave* OR list*)) OR (medical PRE/0 (absen* OR leave*)) OR certified PRE/0 absen* OR (illness NEAR/1 (day* OR absen*)) OR (wORk NEAR/1 absen*) OR re-employ* OR reemploy* OR "back to wORk" OR ((wORk* OR job OR employment) NEAR/1 (return* OR re-ent* OR reent*)) OR ((vocational OR occupational) NEAR/0 (rehab* OR reintegration* OR re-integration*)) OR "suppORted employment" OR (disability PRE/0 (absen* OR allowance* OR benefit* OR insurance* OR leave* OR pension*)) OR (wORk* PRE/0 (compensation* OR resumption*)))) AND (MAINSUBJECT.EXACT("Motivation") OR Ti,AB,SU(motivational PRE/0 interview* OR "motivational enhancement")) AND pd(19830101-20190814)

**Database: SveMed+**

**Date: 14.08.2019**

**Records: 44**

1 noexp:"Motivational Interviewing" 37

2 ("motivational interviewing" OR "motivational interview" OR "motivational interviews" OR "motivational enhancement") 44

3 #1 OR #2 44

**Database: DARE (The Database of Abstracts of Reviews of Effects) & HTA (Health Technology Assessment)**

**Date: 31.01.2019**

**Records: 1**

1 MeSH DESCRIPTOR return to work IN DARE,HTA 15

2 MeSH DESCRIPTOR employment, supported IN DARE,HTA 8

3 MeSH DESCRIPTOR rehabilitation, vocational IN DARE,HTA 28

4 MeSH DESCRIPTOR sick leave IN DARE,HTA 27

5 MeSH DESCRIPTOR absenteeism IN DARE,HTA 20

6 ((sickleave* or sicklist* or (sick* adj (absen* or allowance* or benefit* or certifi* or day* or insurance* or leave* or list*)) or (medical adj (absen* or leave*)) or certified absen* or (illness adj2 (day* or absen*)) or (work adj2 absen*) or re-employ* or reemploy* or "back to work" or ((work* or job or employment) adj2 (return* or re-ent* or reent*)) or ((vocational or occupational) adj1 (rehab* or reintegration* or re-integration*)) or supported employment or (disability adj (absen* or allowance* or benefit* or insurance* or leave* or pension*)) or (work* adj (compensation* or resumption*)))) IN DARE, HTA 242

7 #1 OR #2 OR #3 OR #4 OR #5 OR #6 269

8 MeSH DESCRIPTOR Motivational Interviewing IN DARE,HTA 27

9 (motivational interview*) IN DARE, HTA 111

10 (motivational enhancement) IN DARE, HTA 9

11 MeSH DESCRIPTOR Motivation IN DARE,HTA 121

12 #8 OR #9 OR #10 OR #11 210

13 #7 AND #12 1

**Database: Epistemonikos**

**Date: 14.08.2019**

**Records: 1 Systematic Review**

(sickleave* OR sicklist* OR "sickness absence" OR "sickness absences" OR "sickness allowance" OR "sickness allowances" OR "sickness benefit" OR "sickness benefits" OR "sickness certification" OR "sickness certifications" OR "sick day" OR "sick days" OR "sickness insurance" OR "sickness insurances" OR "sick leave" OR "sick leaves" OR "sick list" OR "sick lists" OR "sick listed" OR "sick listing" OR "sick listings" OR "medical absence" OR "medical absences" OR "medical leave" OR "medical leaves" OR "certified absence" OR "certified absences" OR "illness day" OR "illness days" OR "illness absence" OR "illness absences" OR "work absence" OR "work absences" OR re-employ* OR reemploy* OR "return to work" OR "return-to-work" OR "returning to work" OR "back to work" OR "back-to-work" OR "vocational rehabilitation" OR "vocational reintegration" OR "vocational re-integration" OR "occupational rehabilitation" OR "occupational re-integration" OR "occupational reintegration" OR "supported employment" OR "disability absence" OR "disability absences" OR "disability allowance" OR "disability allowances" OR "disability benefit" OR "disability benefits" OR "disability insurance" OR "disability insurances" OR "disability leave" OR "disability leaves" OR "disability pension" OR "disability pensions" OR "workers compensation" OR "workers’ compensation" OR "work resumption" OR "work resumptions") AND ("motivational interviewing" OR "motivational interview" OR "motivational interviews" OR "motivational enhancement")

REFERENCE SEARCHES

| **Date** | **Article** | **Search method** | **Total hits** | **Studies screened in full text** |
| --- | --- | --- | --- | --- |
| 25.04.19 | Magnussen et. al. 2007 | Cited reference search, Web of science | 18 | Britt et. al. 2018. Motivational Interviewing to Promote Employment. |
| 25.04.19 | Magnussen et. al. 2007 | Screened reference list of article. | 34 | No additional relevant studies |
| 01.05.19 | Park et.al. 2017 | Cited reference search, Web of science | 5 | No additional relevant studies |
| 01.05.19 | Park et.al. 2017 | Screened reference list of article. | 27 | No additional relevant studies |
| 01.05.19 | Gross et.al. 2017 | Cited reference search, Web of science | 3 | No additional relevant studies |
| 01.05.19 | Gross et.al. 2017 | Screened reference list of article. | 38 | No additional relevant studies |

OTHER SOURCES

| **Date** | **Database/web page/other resource** | **Description of search method** | **Studies screened in full text** |
| --- | --- | --- | --- |
| **17.01.19** | The Norwegian Labour and Welfare Administration (NAV) webpages  <https://www.nav.no> | Searched all the different areas of the web pages | No relevant studies |
| **01.05.19** | <https://motivationalinterviewing.org> | Searched all the different areas of the web pages. Found a list made by Miller: “Controlled Clinical Trials Involving Motivational Interviewing”, went through the list. | No additional relevant studies |
| **20.05.19** | Journal: MITRIP, motivational interviewing, training, research, implementation, practice  <http://www.mitrip.org> | Search words:  ‘return to work’, 5 hits  ‘back to work’, 16 hits  ‘vocational rehabilitation’ 0 hits  ‘sick leave’ 1 hit  ‘sick*’, 1 hit  ‘disability’, 0 hits  ‘musculoskeletal disorder*’, 0 hits  ‘supported employment,’ 0 hits | No additional relevant studies |
| **20.05.19** | Google news search:  <https://news.google.com/search?q=%22motivational+interviewing%22&hl=en-US&gl=US&ceid=US:en> | “motivational interviewing”, 100 hits  “motivational interview”, 71 hits | No additional relevant studies |
| **20.05.19** | Google search | “Motivational interviewing and return to work”, looked at page 1-5 from the search (50 first hilts).  One relevant book chapter by Mark P. Jensen: ‘Motivational Interviewing to Enhancing Return to Work’ from ‘Handbook of Return to Work’ (2016)  Looked thorough reference list, no relevant studies found. | No additional relevant studies |
| **24.05.19** | Search of newsletter for MINT trainers: MINT Bulletin/ Motivational Interviewing Newsletter: Updates, Education and Training (MINUET)  [*https://motivationalinterviewing.org/bulletin*](https://motivationalinterviewing.org/bulletin) | Hand searches of all the newsletters that have been published (1994-2009). | Manthey, T. 2009.Training MI in a Vocational Rehabilitation Context.  *MINT Bulletin. 2009;15 (1)* |
| **March- august 2019** | Researchers in the field of MI. | Sent e-mails to the following researchers asking if they knew of relevant ongoing studies, or unpublished studies:  Wiiliam R. Miller, Roger Hagen, Gunnhild Bagøyen, Anne Høiby, Lise Cecilie Kleppe, Blanka Støren-Vazcy, Martin Inge Standal, Vegard Stolsmo Foldal, Douglas P. Gross, Liv Magnussen, Liv Strand, Jan Skouen, Hege Eriksen, Nicolette Sheridan. | No additional relevant studies |

**Appendix II: Excluded papers**

| **Study first author, year, name of study** | **Reason for exclusion** |
| --- | --- |
| **Aasdahl, 2018**  Motivational interviewing in long-term sickness absence: study protocol of a randomized controlled trial followed by qualitative and economic studies | Study protocol. Contacted research team, preliminary results not ready. Results for people with musculoskeletal disorders will not be reported separately |
| **Andersen, 2017**  Positive experiences of a vocational rehabilitation intervention for individuals on long-term sick leave, the Dirigo project: a qualitative study | Wrong participants: mainly people with mental health problems, not musculoskeletal disorders |
| **Becker, 2008**  Effects of Two Guideline Implementation Strategies on Patient Outcomes in Primary Care | Wrong outcome: regular physical activity, not return to work.  Wrong intervention: no description of MI |
| **Britt, 2018**  Motivational Interviewing to promote Employment | Wrong participants: mainly people with mental illness or ex-offenders |
| **Flodgren, 2017**  Motivational Interviewing as a method to facilitate return to work. A systematic review | Wrong participants: studies with people with psychiatric conditions, HIV and drug involved offenders. One study with musculoskeletal population, this study is included in the review |
| **Ipsen, 2014**  Evaluation of an Online Health Promotion Program for Vocational Rehabilitation Consumers | Wrong participants: no description of proportion with musculoskeletal disorders  Wrong outcome: improved health, not return to work |
| **Leahy, 2018**  Promising and evidence-based practices in vocational rehabilitation: Results of a national Delphi study | Wrong participants: experts in the field of vocational rehabilitation, not focus on interventions for people with musculoskeletal disorders |
| **Manthey, 2009**  Training MI in a Vocational Rehabilitation Context | Wrong participants: vocational rehabilitation caseworkers, not focus on interventions for people with musculoskeletal disorders |
| **Manthey, 2011**  Motivational Interviewing and Vocational Rehabilitation: A Review With Recommendations for Administrators and Counsellors | Wrong participants: describes literature relevant to the use of MI in vocational services in general, not related to musculoskeletal disorders |
| **Page, 2014**  Use of Motivational Interviewing to Improve Return-to-work and Work-related Outcomes: A Review | Wrong participants and outcomes: studies with people with psychiatric diagnoses, HIV/AIDS and clinical offenders. Only one study with MSK population. This study is included in the review |
| **Ruggiero, 2012**  An effectiveness evaluation of motivational interviewing as an addition to an internet based health promotion program for vocational rehabilitation consumers | Wrong participants: no description of proportion with musculoskeletal disorders  Wrong outcome: positive health behaviour, not return to work |
| **Secker, 2014**  Employment Support Workers’ Experiences of Motivational Interviewing: Results From an Exploratory Study | Wrong participants: employment workers not primary following-up people with musculoskeletal disorders |
| **Sheridan, 2017**  Evaluating the effectiveness of a return to work programme for adults living in Waikato receiving a ‘sickness’ benefit | Registered trial in Cochrane Central Register of Controlled Trials. Contacted principal investigator to hear if preliminary results were ready. No answer received |
| **Ståhl, 2017**  Process evaluation of an inter organizational cooperation initiative in vocational rehabilitation, the Dirigo project | Wrong participants: managers of Swedish state authorities and project staff in the Dirigo project |
| **Ståhl, 2018**  Introducing Motivational Interviewing in a Sickness Insurance Context: Translation and Implementation Challenges | Wrong participants: Swedish sickness insurance officials, managers, and regional Coordinators not primarily following-up people with musculoskeletal disorders |
| **Thompson, 2012**  Are you ready? Readiness to return to work for people living with chronic pain | Wrong participants: people with different pain conditions. Not primary musculoskeletal disorders |
| **Torres, 2019**  The Impact of Motivational Interviewing Training on Rehabilitation Counsellors: Assessing Working Alliance and Client Engagement. A Randomized Controlled Trial | Wrong participants: vocational rehabilitation counsellors and their clients, mainly people with mental disabilities |
| **Wagner, 2004**  Motivational Interviewing and Rehabilitation Counselling Practice | Wrong type of study: not empirical study, theoretical study, no empirical data |
| **Øiestad, 2019**  Effect of Motivational Interviewing on Return to Work in Sick Listed People With Musculoskeletal Disorders | Registered trial in clinical trial register. Ongoing trial, still recruiting |
